# Living inside the box: environmental effects on mouse models of human disease

**DOI:** 10.1242/dmm.035360

**Published:** 2018-10-01

**Authors:** John P. Sundberg, Paul N. Schofield

**Affiliations:** 1 The Jackson Laboratory, Bar Harbor, ME 04609-1500, USA; 2 Department of Physiology, Development and Neuroscience, University of Cambridge, Cambridge CB2 3EG, UK

**Keywords:** Husbandry, Diet, Environment, Stress, Mouse models, Human

## Abstract

The impact of the laboratory environment on animal models of human disease, particularly the mouse, has recently come under intense scrutiny regarding both the reproducibility of such environments and their ability to accurately recapitulate elements of human environmental conditions. One common objection to the use of mice in highly controlled facilities is that humans live in much more diverse and stressful environments, which affects the expression and characteristics of disease phenotypes. In this Special Article, we review some of the known effects of the laboratory environment on mouse phenotypes and compare them with environmental effects on humans that modify phenotypes or, in some cases, have driven genetic adaptation. We conclude that the ‘boxes’ inhabited by mice and humans have much in common, but that, when attempting to tease out the effects of environment on phenotype, a controlled and, importantly, well-characterized environment is essential.

## Abstract (precis)

**Summary:** The utility of mice as models of human disease is often questioned because they live in a controlled laboratory environment, unlike humans. The authors compare the environments of humans and mice, and find parallels, suggesting that they are not so different. Understanding how mouse and human environments affect phenotypes is critical for the development of models of human disease.

“*Little boxes, little boxes, little boxes all the same*”

– *Pete Seeger's cover of Malvina Reynolds’* ‘*Little Boxes*’^1^

## Introduction

One question we often find ourselves asked is how can mice be realistic models for human diseases when they live in a box? The implication is that mice in laboratories have low psychological, metabolic and immunological stress levels compared with humans. This assumption is based on the concept that mice live in an environmentally controlled cage (a box) with free access to high-quality water (acidified or chlorinated tap water), without pathogens (specific-pathogen-free status), and *ad libitum* access to highly palatable and nutritious feed (also without pathogens as the food is usually pasteurized or sterilized). Laboratory mice live in a room that is environmentally controlled for temperature, humidity, light cycle and contaminants, as air is HEPA (high efficiency particulate air) filtered (Table 1). Aside from encounters with cage-mates and investigators, they are safe from predation. Lastly, mice usually have high-quality medical (veterinary) care. Fundamentally, laboratory mice live in ‘the comparatively plush environment of the shoebox cage’ (Harper, 2008). Some have ‘doubted that commercial lab mice – selectively bred in sanitized environments – are good research analogues for people, who do not live in such clean conditions’ (Reardon, 2016; Beura et al., 2016). The phenotypes that researchers are interested in measuring and understanding are the consequence of a genotype expressed in a particular environment, and a repeatedly made argument is that mice in such a controlled environment cannot be expected to show the same phenotypes as humans even if the underlying genetics are the same. This assumes that there are huge differences between the manner in which mice are raised and how humans live.

**Table 1.**
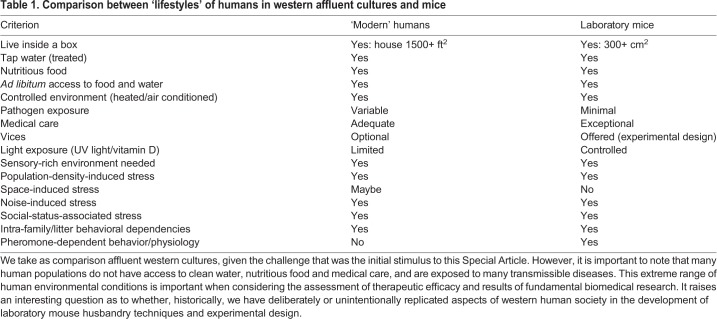
**Comparison between ‘lifestyles’ of humans in western affluent cultures and mice**

However, when one is at the podium in a lecture hall it becomes obvious that the human population (the audience) is, in fact, living within a box (the lecture hall and, by extension, modern western homes and offices). These housing facilities are temperature controlled, air is ventilated and filtered, and lighting is controlled. Food is readily available (coffee, tea and cookies at the back of the lecture hall) and heavily regulated to ensure high nutritional quality and low to no pathogen exposure. Likewise, water from public sources is also highly controlled, frequently monitored for quality and treated to eliminate pathogens, and is readily available (*ad libitum* food and water). Medical care is, for the most part, readily available and of high quality. While these are generalities, they do represent how the majority of people live in developed countries.

## Life in nature's ‘box’

Mice and humans are both remarkably adaptable animals and can be found in a huge range of environments, showing adaptive responses to overcrowding, food and shelter availability, temperature and predation (Silver, 1995).

“*The planet-wide spread of the house mouse in both man-made and natural habitats suggests an extreme reproductive adaptability, probably the most extreme among the mammals. Only humans are as adaptable (some would say less so)*.” *(**Bronson, 1984**)*

Much of the argument against using laboratory mice as models rests on the tacit assumption that there is a ‘normal’ environment for both humans and mice (Beura et al., 2016) – a ‘state of nature’ – and that, for mice, the laboratory environment is far removed from it, but that humans somehow live in a ‘natural environment’. This concept is self-evidently flawed, largely because, during human evolution, we have taken control of our own environment to a great extent, or evolved very specific behaviors or genetic traits to deal with it. For example, the evolution of skin pigmentation in hominins is accepted to have been dependent on the latitude at which populations live (Marciniak and Perry, 2017). Probably driven by the requirement for vitamin D synthesis, northern populations have lost their ancestral dark pigmentation. An exception to this seems to be the Inuit in the American Subarctic and far Northeast Asia, who have darker skin pigmentation than others living at the same latitude, such as Swedish populations. One hypothesis is that retention of darker pigmentation is a response to the requirement to protect from high UV exposure during the long and intense daylight periods of the arctic summer, but this is compensated for by a diet that is very rich in vitamin D (Schaebel et al., 2015). Concomitant selection for genetic variants associated with this diet and environment (Fumagalli et al., 2015) shows that this population has become extremely adapted both behaviorally and genetically. So the ‘box’, the environment in which the Inuit live, is very different from the ‘box’ that biological scientists in Maine or lawyers in Manhattan inhabit.

A parallel story for the mouse exists. Over the past 15,000 years, the genus's close association with human beings has significantly affected its evolution. There is now clear evidence that commensal species of mice began living with humans at the time when humans became sedentary, and the anthropogenic environment created provided selective advantages for sympatric and commensal species of mice (Weissbrod et al., 2017). This close association has had demonstrable effects on phenotypes such as coat color, skull morphology and possibly behavioral traits associated with living in close proximity to humans and the other commensal animals under domestication at the time or earlier, such as dogs (Botigué et al., 2017) and cats (Ottoni et al., 2017). As we discuss above, the mouse has colonized many habitats, with *Mus musculus domesticus* being the species most closely influenced by the human ‘box’. However, the reader must remember that the current strains of laboratory mice have also been bred from other species that have been associated in different ways and for different time periods with humans. The resulting range of laboratory strains available thus samples and recombines traits that evolved in quite different environments. Laboratory strains are best regarded as recombinants derived from three ancestral components: *M.* *musculus domesticus*, *Mus musculus musculus* and *Mus musculus castaneus* (Guénet and Bonhomme, 2003). The collaborative cross is now helping create further reassortment of the genetic variation available in inbred strains (Srivastava et al., 2017).

## Life in the laboratory box

The idea that laboratory mice have been selected over a century for traits associated with both husbandry, such as fast maturation and ease of handling, and with those of specific scientific interest, such as predisposition to the diseases for which they are used as models, is perhaps expected. However, strains differ considerably in their ‘wildness’ and aggression (Wahlsten et al., 2003). Some commonly used strains, such as SPRET/EiJ, are notoriously difficult to handle. The AKR strain, which shows a high incidence of leukemia, has a very high degree of intra-strain aggression (Simon, 1979). AKR was derived from a pet shop stock in the 1920s and SPRET/EiJ from wild captured *Mus spretus* stock from Spain in the late 1970s. It is unclear whether aggression was co-selected during the active selection for the desired strain characteristics in the research laboratory environment, or whether it was retained in these and other strains. However, it is quite evident that rearing in a laboratory environment does not automatically result in selection for docile mice.

An example of laboratory selection from wild-type variation is that of the progressive retinal degeneration phenotype associated with the *rd1* allele (*Pde6b^rd1^*), first discovered in CBA mice and then as independent spontaneous mutations in C57BL/6 and C3H mice (Farber et al., 1994). These mice are used as a model for human retinal degenerative diseases such as autosomal recessive retinitis pigmentosa (OMIM:180072). Retinal degeneration mutations are found in many mouse strains, but it is unclear whether these have been selected by the laboratory environment itself or whether the environment is simply permissive for their spread through laboratory strains. It would seem likely that mice carrying these mutations would not have survived in the wild. However, they have been found in some recent wild-derived inbred strains, implying that the mutations may be present in some wild populations (Chang et al., 2002). Retinal degeneration mutations are similarly relatively common in the human population, with 1 in 2000-3000 individuals having some form of genetic retinal degenerative disease (Veleri et al., 2015).

## Environmental carcinogens

**Fig. 1.**
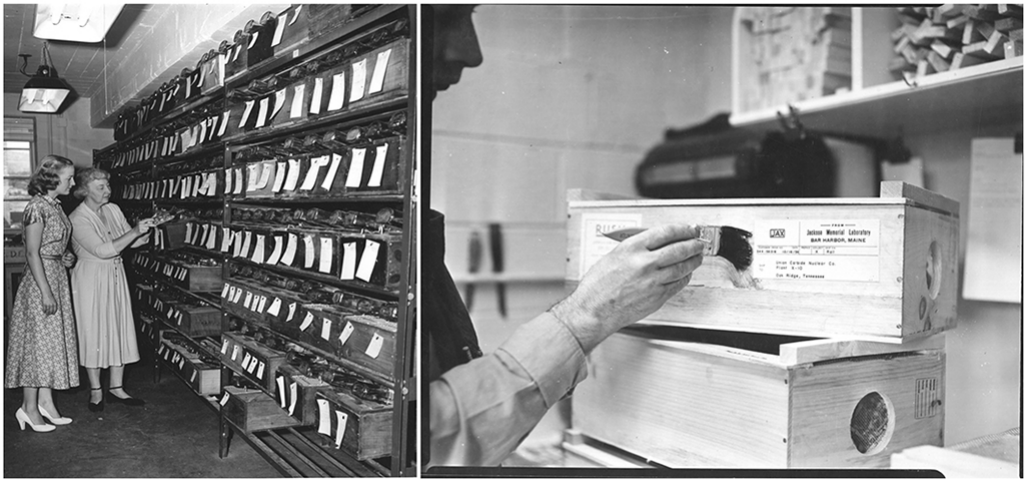
**Historical development of the mouse ‘shoe box’.** Mouse facility at The Jackson Laboratory in the 1950s, showing racks containing the original double-sided wooden mouse boxes. The original wooden boxes were modifications of boxes used by the Maine blueberry industry to transport berries. In the early days, mice were shipped in wood containers with hardware cloth covering openings on the sides for aeration. Source: The Jackson Laboratory Archives.

Life in the box, whether mouse or human, has not always been as snug as people think. While we take this for granted, current laboratory animal husbandry practices were developed largely by trial and error. For example, mouse boxes evolved out of ‘shooks’, wooden boxes used to transport wild blueberries in Maine (E. Less, personal communication; see Fig. 1). The lumber was readily available in precut sizes, making it cheap and easy to create the boxes. To make them last, the wood was coated with creosote, but the mice then developed a high incidence of cancer (Boutwell and Bosch, 1958; Holme et al., 1999; Holstein, 1979; Schoket et al., 1988). This was not surprising, as a similar correlation was made nearly 200 years earlier for chimney sweep boys, who developed a high incidence of scrotal squamous cell carcinoma (Torronen et al., 1989; Waldron, 1983). These wooden boxes were later replaced by stainless steel and, currently, by plastic boxes. A wide variety of bedding types continue to be used to house mice in these boxes. The different bedding types can affect breeding (Iturrian and Fink, 1968; Jackson et al., 2015; Tanaka et al., 2014), overall health (Becker et al., 2010; Ferrecchia et al., 2014; Horn et al., 2012; Potgieter and Wilke, 1996) or metabolism. This can lead to a wide variety of disorders, including cancer (Jacobs and Dieter, 1978; Li et al., 2009; Nielsen et al., 1986; Schoental, 1974; Torronen et al., 1989; Vesell, 1967; Vlahakis, 1977). Living inside the box can lead to a variety of health problems in humans as well (Bond, 1966; Ritchie, 1946; Williams, 1948; Wilner et al., 1958).

## Diet

Diet can have a profound effect on the phenotype of mice. One of the most studied dietary issues is the effect of high-fat diets on the development of atherosclerosis. Mice, normally fed a low-fat vegetarian-based diet, rarely if ever develop spontaneous atheromatous plaques in their arteries. Atherogenesis can be induced by placing the mice on high-fat diets, especially for lines that carry mutations involved in the pathogenesis of this disease (Chistiakov et al., 2017). Diet can modify the onset and severity of disease as well; for example, ectopic soft-tissue mineralization, a feature of pseudoxanthoma elasticum (Uitto et al., 2014) and several related diseases (Li and Uitto, 2013), can be exacerbated when mice are fed an experimental diet high in phosphate and low in magnesium content (Jiang and Uitto, 2012; Li et al., 2015). This so-called ‘acceleration diet’ shortens the time the investigated mice require to develop features of pseudoxanthoma elasticum. Consequently, adopting dietary modifications for humans with this condition are currently under investigation (https://clinicaltrials.gov/ct2/show/NCT01525875; Renie et al., 1984; Sherer et al. 2005).

Obesity and type 2 diabetes are a global problem in human populations. This disease has been recapitulated in mouse models, especially when using standardized high-fat diets (Heydemann, 2016). Similar to humans, there are clear interactions between diet and exercise in mice (Nogueira et al., 2017), with different exercise regimes, such as swimming or treadmill running, ameliorating the physiological and pathological effects of a high-fat diet. Exercise-associated cardiac hypertrophy is also seen in both mice and humans (Evangelista et al., 2003). Interestingly, like humans, different strains of mice show different compliance levels with exercise regimes and, at the risk of sounding anthropomorphic, it is tempting to compare treadmill exercise avoidance behavior in C57BL/6 with our own human experience (Gibb et al., 2016).

One can argue that laboratory mice do not have access to human ‘vices’ or hedonic behaviors – rewarding stimuli gained through behavior – particularly those associated with dietary intake of types of food, alcohol and narcotics (Jimenez et al., 2017). In fact, mice are widely used for studies on voluntary self-administration of alcohol (Mayfield et al., 2016) and chocolate (Patrono et al., 2015), and have been shown to ‘self-medicate’ when repeatedly exposed to stressors (Mollenauer et al., 1993). So, while we limit access to such ‘vices’ by forced confinement, mice can have access and do take advantage of these opportunities, when available, just as humans do. It is of interest that feral mice will actively choose to wheel run in their natural environment when presented with a wheel and no other obvious reward (Meijer and Robbers, 2014), and seem to find the activity rewarding in itself.

## Immunity and environmental pathogen exposure

There is evidence that the immune system of wild-living mice differs from that of laboratory strains, being highly activated and reflecting the constantly changing and heavy burden of pathogens to which the former are exposed. Their innate immune systems are however remarkably similar, with the exception that myeloid cells from wild-type mice do not seem to mount as aggressive a cytokine response *in vitro* as those from laboratory mice, suggesting selection for relative damping of the response in the wild (Abolins et al., 2017), and a novel population of myeloid cells seems to be absent from laboratory strains. The activity of the mouse adaptive immune system has been shown to change depending on early exposure to various pathogens (Tao and Reese, 2017). For example, co-housing with ‘pet shop’ mice, which are derived from free barn-living ‘feral’ populations, was shown to change the immune profile of laboratory mice, modifying it to one more closely resembling the adult human, with concomitant increased resistance to infection and altered T-cell kinetics (Beura et al., 2016). However, human populations do not all experience the same antigenic and disease environment, and this has wide-reaching consequences for the immune system (Grignolio et al., 2014). Transmissible disease accounts for about half of premature (<45 years) deaths in the developing world. In the developed world, sanitation and healthcare have radically reduced the number of pathogens to which humans are exposed (Guarner et al., 2006). Changes in lifestyle and environmental exposure, healthcare and antibiotic use have had profound effects on the human microbiome (Bloomfield et al., 2016), one negative consequence of which has been the increase in allergic and autoimmune disease, and possibly some types of neoplasia, such as acute lymphoblastic leukemia (ALL) (Bach, 2017; Greaves, 2009, 2018).

The microbiome, the large variety of microorganisms living in and on everyone and everything, can modulate host phenotypes no matter how carefully we attempt to control them. Spontaneous inflammatory bowel disease (IBD) was first identified in mice nearly 25 years ago (Sundberg et al., 1994). It was later shown to be modulated by *Helicobacter hepaticus*, an opportunist bacterial species originally thought to be normal flora and thus unimportant (Cahill et al., 1997; Foltz et al., 1998; Ward et al., 1994). Modifications in the microbiome are now known to affect a wide variety of phenotypes in mice (Franklin and Ericsson, 2017; Su et al., 2016; Zachariassen et al., 2017) and humans (Bohn et al., 2017; Kuk et al., 2016; Lloyd-Price et al., 2016). So, just as in mice, we know that the antigenic environment in which we live affects our health, but this is different for different populations. Again, we find that humans live in ‘boxes’, but there are many different ones.

## Effects of environment on ethologically appropriate behaviors

There has been much discussion about how to make a laboratory mouse ‘happy’ (Grimm, 2018) by enriching its environment. Attempts to assess mouse ‘happiness’ are subjectively anthropomorphic. Nevertheless, a reduction of stress and support for richer behaviors certainly produces a more ethologically appropriate set of behaviors and more normal physiology. There are many instances in which such environmental enrichment changes the outcome of experiments, and the association with a sensory-rich environment affects disease susceptibility and outcomes in human beings (Grimm, 2018). Manipulation of the cage environment can also be used to manipulate behaviors, and a wide variety of stressors can be added to a box cage for a mouse, such as turning a spotlight on and off (Aarts et al., 2015) or noise (Munzel et al., 2017), both of which are known physiological stressors in humans.

In addition to enrichment and cage size itself, pheromonal cues and housing density are all well-characterized factors that affect reproductive phenotypes. These factors are becoming a greater concern with the increasing use of ventilated cages, where pheromones cannot accumulate to any significant degree. It has long been known that putting an unknown (‘strange’) male or female in a box with another male or female will produce endocrine changes in both sexes, affecting behavior and physiology (Macrides et al., 1975; Nichols and Chevins, 1981). While only a few inbred strains are used in most of these studies, those that use more than one strain find that the responses vary between them (Heinla et al., 2018). Pheromones from urine or vaginal sites can modulate ultrasonic courtship in male mice (Nyby et al., 1977), and male urine can affect sexual maturation in females (Coppola and Vandenbergh, 1985), presumably via pheromones released by the preputial and clitoral glands. To improve the success of timed matings when female and male mice are housed together for a limited time, breeding pairs can be maintained in the same box separated by a transparent partition for 3 days before setting up the timed mating. This permits male urine-borne pheromones to induce female estrus, thereby enabling the expression of male and female mating behaviors (Stiles et al., 2013).

These and related observations have raised concerns about sanitation practices in mouse husbandry that can alter pheromonal signaling and potentially cause confounding effects in the research. Even the organization of a mouse room, segregating male and female mice, could have a significant effect on experimental results (Bind et al., 2013; Lloyd et al., 2018). Evidence regarding the effects of human pheromones is highly controversial, but a recent paper suggests that this is one area where mice and humans might have rather distinct environmentally mediated communication mechanisms (Hare et al., 2017).

The cage environment also affects other aspects of reproduction. Parental interactions, both with pups and between mothers and fathers, are highly significant for both mice and humans (Glasper et al., 2018; Kentner et al., 2010; Liu et al., 2013), and there is an increasing awareness of the importance of paternal interactions within the family or litter environment in both species.

The effect of housing and population density have complex but non-identical effects on mice and humans. Caging systems can affect the health and behavior of mice (Polissidis et al., 2017), although adverse physiological effects at stocking densities up to three times the recommended levels have been difficult to identify (Paigen et al., 2012; Morgan et al., 2014). The urban environment is reported to have a significant effect on human health and wellbeing. For example, noise and overcrowding (Park and Evans, 2016), and social interactions and status, all impact human disease susceptibility and wellbeing (Fassio et al., 2012; Lederbogen et al., 2011). On a species basis, however, it is difficult to assess and compare the relative severity of the physical environments to which humans and mice are exposed. Does 10 people living in a single room equate to 16 mice per shoebox cage with 31 cm^2^ each?

Although it is difficult to disentangle the different elements of human urban life, the issues of housing space and population density are interesting. Group size and composition clearly affect the behavior of mice (Kappel et al., 2017; Lee et al., 2018), and crowded male mice of several strains show high levels of aggression. However, contrary to much received wisdom, the most important positive factor seems to be a rich physical and social environment, and not simply the space available, at least within the constraints of a laboratory setup (Bailoo et al., 2018). This is mirrored by a recent human study where, surprisingly, the size of houses *per se* has rather little effect on several indicators of overall subjective wellbeing (Foye, 2017); this is a consideration that is becoming more important in the UK particularly, which now has the smallest new housing stock in Western Europe (Morgan and Cruikshank, 2014). The question we need to ask is: how do we take into account this range of human environmental effects in clinical studies, just as we would with mice? In many ways, the impacts on ethologically appropriate behaviors of running a mouse experiment in a laboratory environment are much better recognized than those of the human environment in therapeutic clinical trials.

## Heterogenization and population pharmacokinetics

One way of dealing with the unknown effects of environment – the ‘unknown unknowns’ – on experimental measurements or outcomes is to use data from a wide variety of randomly assigned or naturally occurring environments, or even genotypes. Embracing variability has the potential to make a study more reproducible and externally valid. This may either be done with systematized heterogenization, as proposed by Richter et al. (2010, 2009), or by combining studies conducted at different research sites (Kafkafi et al., 2005). Recently, Voelkl and colleagues (2018) reported simulated multi-laboratory studies where genetic and environmental variability, different strains, ages, housing conditions, etc., were used. This approach clearly has merits and it will be interesting to see how it might improve reproducibility in physiological, as well as behavioral, studies, where most effort has so far been focused (Karp, 2018). Interestingly, a similar strategy was developed for population pharmacokinetic studies in humans in the 1980s following concerns that the pharmacokinetics of new drugs were not being studied in relevant populations (Aarons, 1991). In this approach, data from all individuals in a population are evaluated simultaneously using nonlinear mixed-effects modeling (Mould and Upton, 2013). Such approaches actually depend on humans and mice living in a variety of boxes and the heterogeneity thus incorporated into the study can, in principle, make results much more robust.

## Cauda

The challenge of investigating the impact of environment on phenotype is considerable, but to approach it by using ‘dirty’ mice in a stressful environment complicates the separation of the impact of any single aspect of the environment from another. The situation is similar when trying to establish the environmental impact on disease in humans. As we cannot put patients into a mouse house, we have to carry out demographic, ecological, toxicological and genetic studies to establish environmental impact. For both humans and laboratory mice, we argue that accurate recording, capture and characterization of environmental parameters is key to understanding how they affect phenotypic expression. Much effort was made in the European Mouse Disease Clinic (EUMODIC) and International Mouse Phenotyping Consortium (IPMC) to standardize assays and capture formal environmental metadata for phenotype assays carried out at multiple sites across the world. This has provided objective assessments of the impact of the experimental environment on reproducibility and insights into the interactions between phenotype and environment (de Angelis et al., 2015). This is inevitably a bootstrapping exercise that requires both mouse and human studies to move in lock-step towards a model replicating all or at least some of the human disease. As Robert Koch said, “remember gentlemen, a mouse is not a human being” (Greep, 1970). But, with renewed awareness of the importance of controlling and understanding the laboratory environment and its relationship to human environments, the mouse continues to be a powerful model.

## Footnotes

1 Original words copyright 1962 Schroder Music Company (https://people.wku.edu/charles.smith/MALVINA/mr094.htm).
